# Data on effect of mulches on growth and fruit yield of watermelon (*Citrullus lanatus* Thunb.) varieties in west Dembia district, central Gondar zone, Ethiopia

**DOI:** 10.1016/j.dib.2024.110071

**Published:** 2024-01-17

**Authors:** Getahun Yismaw, Solomon Fantaw, Asrat Ayalew

**Affiliations:** Department of Horticulture, College of Agriculture and Environmental Sciences, University of Gondar, Gondar, Ethiopia

**Keywords:** Dataset, Growth, Mulches, Varieties, Watermelon, Yield

## Abstract

Watermelon is an important horticultural crop which is grown in warm climate worldwide. However, its production and productivity is low owing to lack of high yielding improved varieties and poor knowledge of using mulches. Therefore, a field experiment was conducted in west Dembia district under irrigation from January to April 2021 to investigate the effect of mulches on growth and fruit yield of watermelon varieties. Factorial combinations of two varieties of watermelon (Crimson Sweet and Sugar Baby) and four types of mulches (black plastic, white plastic, grass mulch and no mulch as control) were arranged in a randomized complete block design with three replications. The remaining necessary agronomic practices and crop management activities were undertaken uniformly. The data presented under this dataset article includes phenological parameters (i.e. Days to 50 % germination, Days to 50 % flowering, and Days to 50 % maturity), growth parameters (i.e. main vine length, number of lateral branches per vine, number of nodes on main vine, and number of leaves on the main vine) and yield and yield component parameters (i.e. Number of total fruit plant^−1^, number of marketable fruit plant^−1^,number of unmarketable fruit plant^−1^, fruit length, fruit diameter, average fruit weight, marketable fruit yield, unmarketable fruit yield and total fruit yield). All the collected data were subjected to analysis of variance (ANOVA) and the analysis was carried out using the SAS version 9.4 software computer program's General Linear Model (GLM) procedure [1]. As described in Montgomery [2], the residuals were examined to verify the normal distribution and homogeneous variance model assumptions on the error terms for each response variable. Because the eight treatment combinations were randomized within each block, the independence assumption is valid. When a treatment effect was significant, multiple means comparison was performed at a 5 % level of significance using the least significant difference (Fisher's LSD) method to generate letter groupings and correlation analysis was performed using the Pearson correlation procedure found in SAS. This dataset article, therefore gives information about the effects mulching on productivity of watermelon varieties. Additionally, it provides the appropriate and economically feasible type of mulching material for maximized fruit yield of watermelon varieties in the study area or other areas having similar agro ecology. Hence, this information can allow other researchers to review the supplement data, methods, and make detailed analysis, which possibly giving rise to new lines of inquiry. This can also give rise to new collaborations and boost the reputation of the present research data within the scientific community and to make it available to everyone around the subject matter to use as they wish.

Specifications Table

**Table:** 

Subject	Agriculture
Specific subject area	Horticulture
Type of data	Table and Figures
How the data were acquired	Data of watermelon related to phenological, growth, yield and yield component parameters of watermelon were collected by using measurement under field conditions at plant and plot basis based on the nature of the parameter collected.
Data format	Analyzed mean data and raw data
Description of data collection	*Days to 50 % germination, Days to 50 % flowering, Days to 50 % maturity, main vine length, Number of lateral branches per vine, Number of nodes on main vine, number of leaves on the main vine yield, Number of total fruit plant^−1^, Number of marketable fruit plant^−1^, Number of unmarketable fruit plant^−1^, Fruit length, Fruit diameter, Average fruit weight, Marketable fruit yield, Unmarketable fruit yield and Total fruit yield were obtained as described in this article.*
Data source location	*University of Gondar, Central Gondar, Ethiopia, is the owner of the data presented in this article. The experimental sites is located at longitude of 12° 14′ 25′′North and 37° 18′ 3′′ East with an altitude of 1800 m above sea level*[3]*.*
Data accessibility	Raw data were deposited at repository Mendeley data; Dec 29, 2023 Direct URL to data: https://data.mendeley.com/datasets/smfxnxczwd/1

## 1 Value of the Data (Mendeley data, V1, doi: 10.17632/smfxnxczwd.1**)**

➢ Despite the boom in demand of watermelon in Ethiopia, its nutritional importance and high potential and asset for cultivation of watermelon particularly in the west Dembia district as it is endowed with suitable agro-ecology, there is little information on its production and cultural practices and industrial application. Hence, it's not grown commercially in many parts of our country. Ethiopia has a high potential for production of almost all horticultural crops. Lack of information, lack of improved varieties, poor cultural practices and the prevalence of pests are the major contributing challenges for such low productivity in many horticultural crop productions including watermelon.
➢ Moreover, lack of widely adaptive improved varieties and inappropriate agronomic practice are limiting factors and farmers are complaining of difficulty in management activities to obtain high quality yield. Hence, there were variations in different varieties in their growth, yield and quality responses. In addition, the use of mulching is an important cultural practice that contribute to have higher yield and quality by controlling the water loss, change in temperature, protecting the fruits from direct contact of the soil and decaying and also general wellbeing of the crops.
➢ However, farmers in the study area did not use mulch and there is paucity of information in respect to use of well adaptive variety in the country as well as the study area. The use of mulching for watermelon production was not well studied and less practiced in Ethiopia. Also the response of watermelon varieties for different type of mulch is not clearly determined in west Dembia district. But, the application of appropriate field cultural management practices and the choice of varieties are the two factors that affect the productivity and quality of watermelon.
➢ Thus, production and expansion of the crop could be enhanced through systematic investigation that focuses on use of mulches and variety combination to come up with concrete recommendation for increasing growth and fruit yield. As a result, this data set will be valuable for researchers, extension workers and farmers in choosing appropriate watermelon variety and mulching type for optimal and sustainable production in the study area and similar agro-ecology.

## 2 Background

In Ethiopia, the market demand of watermelon becomes higher from time to time specially by community in and around cities since people gradually aware of its nutritive and economic importance. Likewise the number of farmers engaged in the production of watermelon and the area of production increased. However, the quality that has been produced by Ethiopian farmers is still very low compared to the word standard and also the productivity is low due to various factors. Among the factors that affecting growth and yield of watermelon are the genetic nature of the varieties, growing environment, insect pest and disease management and cultural practices are some.

Even though, improved variety and mulching are commonly used for watermelon production in most of the producing countries, it is not widely used in Ethiopia. Therefore, growers are in dire need to increase the production, productivity and quality to meet the potential market demands. As a result it is mandatory to improve its productivity with supplement of different agronomic practices and identifying adaptable varieties for a study area.

## 3 Data Description

**Fig. 1:**
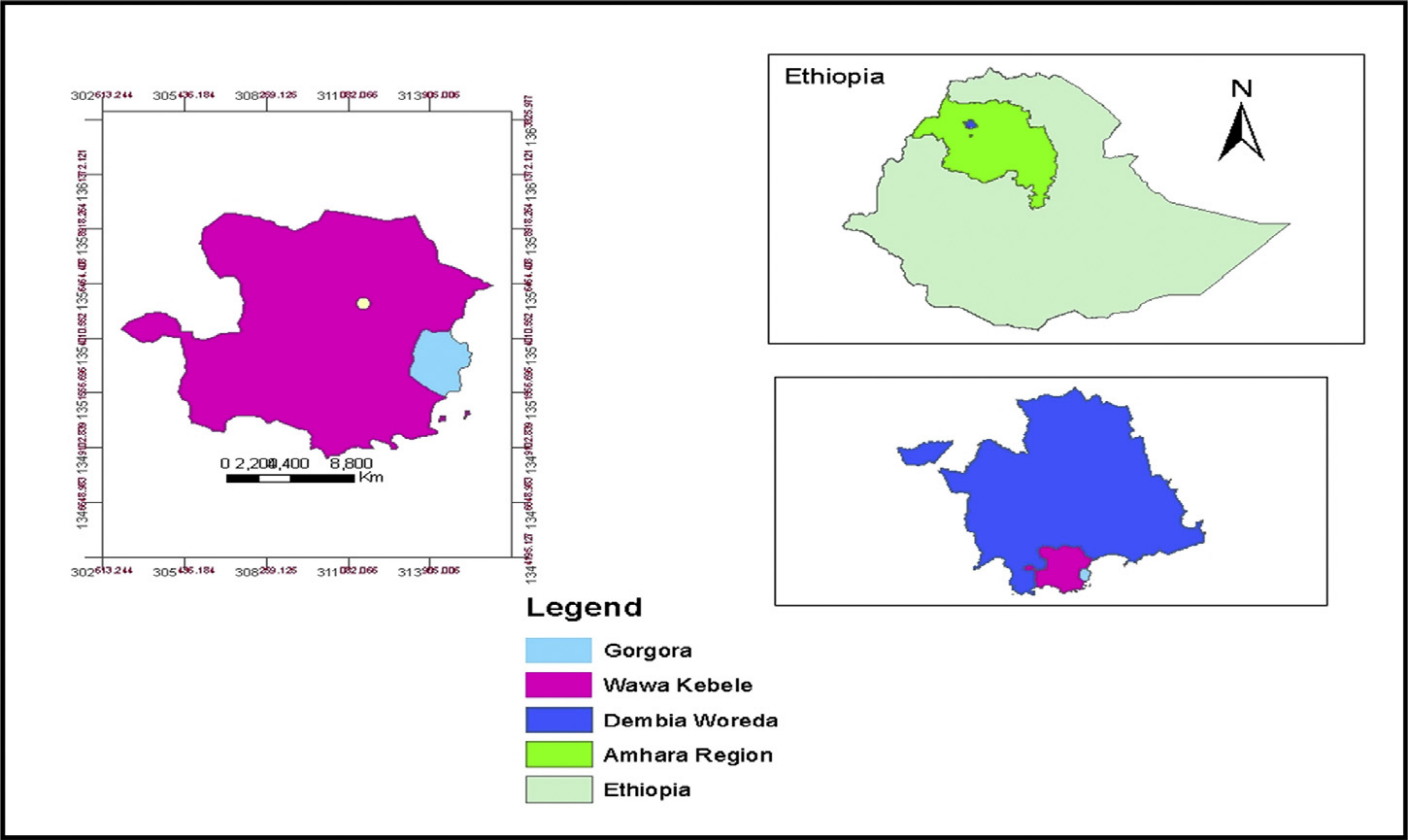
Geographical location of the study area, Wawa kebele, West Dembia, Ethiopia.

This dataset was collected in a field experiment conducted under irrigation from January to April 2021 at Wawa village of West Dembia district, Central Gondar Zone, Ethiopia (Fig. 1). Table 1 illustrates the main effect means of watermelon crop phenology (i.e. Days to 50 % germination, 50 % flowering, and 50 % maturity) parameters as affected by mulches and varieties. The interaction effect means of lateral branches per vine and main effect means (i.e. main vine length (cm), number of leaves on main vine and number of nodes on main vine) of watermelon as affected by mulches and varieties are indicated in Tables 2 and 3 respectively. The main effect means of yield and yield component parameters (i.e. fruit length, fruit diameter, number of marketable fruit plant^−1^ and unmarketable fruit yield) of watermelon as affected by mulches and varieties are indicated in Table 4. The interaction effects of mulches and varieties on number of unmarketable fruit plant^−1^, number of total fruit plant^−1^, average fruit weight, marketable fruit yield and total fruit yield of watermelon are presented in Table 5, Table 6, Table 7, Table 8, Table 9. The dataset presented in this article shows that, fruit yield of watermelon was significantly and positively correlated with most of the growth and yield parameters as shown in Pearson's correlation matrix in Table 10. The partial budget analyzed dataset for fruit yield watermelon as influenced by mulches and varieties is also presented in Table 11. The raw data for parameters collected are available in the repository DOI: 10.17632/smfxnxczwd.1

**Table 1:** Main effect means of watermelon crop phenology as affected by mulches and varieties.

Treatments	Days to germination	Days to flowering	Days to maturity
Mulches			
Control	12.50^a^	55.50^a^	83.33^a^
Grass	12.17^a^	48.67^b^	81.16^b^
White plastic	7.33^b^	43.66^c^	78.16^c^
Black plastic	7.33^b^	44.83^c^	75.33^d^
LSD(0.05)	1.01	2.22	1.78
Varieties			
Sugar Baby	10.75^a^	48.42^a^	78.58^b^
Crimson Sweet	8.92^b^	46.42^b^	80.42^a^
LSD(0.05)	0.72	1.57	1.26
CV (%)	8.34	3.78	1.81

Means followed by the same letters in a column are not significantly different from each other according to the LSD test at 5 % level of significance.

**Table 2:** Means of lateral branches per vine of watermelon as affected by the interaction effect of mulches and varieties.

Treatments	Number of lateral branches per vine	
Mulches	Varieties	
	Crimson Sweet	Sugar Baby
Control	13.03^b^	10.07^d^
Grass	14.07^b^	11.57^c^
White plastic	17.30^a^	17.80^a^
Black plastic	17.60^a^	16.53^a^
LSD(0.05)	1.32	
CV (%)	5.06	

Means followed by the same letters in a column and rows are not significantly different from each other according to the LSD test at 5 % level of significance.

**Table 3:** Main effect means of main vine length, number of leaves on main vine and number of nodes on main vine of watermelon as affected by mulches and varieties.

Treatments	Main vine length (cm)				Number of leaves on main vine			Number of nodes on main vine
	30 DAS	45 DAS	60 DAS	Harvest	30 DAS	45 DAS	60 DAS	
Mulches								
Control	34.00^c^	55.87^c^	121.70^d^	181.27^c^	3.30^c^	6.40^b^	13.18^c^	22.23^c^
Grass	36.20^c^	63.07^c^	136.43^c^	202.72^b^	3.63^c^	7.80^b^	17.28^b^	24.37^b^
White plastic	77.40^a^	141.40^a^	217.77^a^	241.37^a^	6.30^a^	13.46^a^	22.46^a^	26.27^a^
Black plastic	59.70^b^	110.03^b^	193.03^b^	224.67^a^	5.27^b^	12.37^a^	21.37^a^	25.57^ab^
LSD(0.05)	11.01	15.89	11.64	19.9	0.81	2.07	2.43	1.59
Varieties								
Sugar Baby	45.57^b^	78.57^b^	147.63^b^	187.15^b^	4.12^b^	8.97^b^	17.47^b^	22.98^b^
Crimson Sweet	58.08^a^	106.62^a^	186.83^a^	237.86^a^	5.13^a^	11.04^a^	19.68^a^	26.23^a^
LSD(0.05)	7.80	11.24	8.23	14.07	0.57	1.46	1.72	1.13
CV (%)	17.19	13.86	5.62	7.56	14.12	16.69	10.59	5.23

Means followed by the same letters in a column are not significantly different from each other according to the LSD test at 5 % level of significance.

**Table 4:** Main effect means of fruit length, fruit diameter, number of marketable fruit per plant and unmarketable fruit yield of watermelon as affected by mulches and varieties.

Treatments	Fruit length (cm)	Fruit diameter (cm)	NMFPP	UMFY(t ha^−1^)
Mulches				
Control	18.06^c^	17.49^b^	7.32^c^	7.89^a^
Grass	19.48^b^	17.76^b^	8.00^b^	6.00^ab^
White plastic	20.91^a^	19.93^a^	8.52^ab^	3.92^b^
Black plastic	21.96^a^	20.16^a^	8.88^a^	3.99^b^
LSD(0.05)	1.09	1.28	0.54	2.50
Varieties				
Sugar Baby	18.48^b^	17.59^b^	8.13	5.97
Crimson Sweet	21.73^a^	20.08^a^	8.22	4.93
LSD(0.05)	0.77	0.90	ns	ns
CV (%)	4.39	5.48	5.31	37.12

Where: NMFPP= Number of marketable fruit per plant, UMFY=Unmarketable fruit yield and ns= non-significant.

Means followed by the same letters in a column are not significantly different from each other according to the LSD test at 5 % level of significance.

**Table 5:** Means of number of unmarketable fruit per plant of watermelon as affected by the interaction effect of mulches and varieties.

Treatments	Number of unmarketable fruit per plant	
Mulches	Varieties	
	Crimson Sweet	Sugar Baby
Control	3.07^abc^	3.43^a^
Grass	2.70^abc^	3.37^ab^
White plastic	3.37^ab^	2.50^c^
Black plastic	2.63^bc^	2.67^bc^
LSD(0.05)	0.76 14.70	
CV (%)		

Means followed by the same letters in a column and rows are not significantly different from each other according to the LSD test at 5 % level of significance.

**Table 6:** Means of number of total fruit per plant of watermelon as affected by the interaction effect of mulches and varieties.

Treatments	Number of total fruit per plant	
Mulches	Varieties	
	Crimson Sweet	Sugar Baby
Control	10.23^d^	10.40^bcd^
Grass	10.47^cd^	11.60^abc^
White plastic	12.30^a^	10.60^bcd^
Black plastic	11.67^ab^	11.40^abcd^
LSD(0.05)	1.17 6.01	
CV (%)		

Means followed by the same letters in a column and rows are not significantly different from each other according to the LSD test at 5 % level of significance.

**Table 7:** Means of average fruit weight of watermelon as affected by the interaction effect of mulches and varieties.

Treatments	Average fruit weight (kg)	
Mulches	Varieties	
	Crimson Sweet	Sugar Baby
Control	4.50^c^	4.83^c^
Grass	5.68^bc^	5.36^c^
White plastic	6.95^ab^	5.71^bc^
Black plastic	7.91^a^	5.39^c^
LSD(0.05)	1.43	
CV (%)	14.12	

Means followed by the same letters in a column and rows are not significantly different from each other according to the LSD test at 5 % level of significance.

**Table 8:** Means of marketable fruit yield of watermelon as affected by the interaction effect of mulches and varieties.

Treatments	Marketable fruit yield (t ha^−1^)	
Mulches	Varieties	
	Crimson Sweet	Sugar Baby
Control	28.78^d^	30.96^cd^
Grass	43.57^b^	37.71^bcd^
White plastic	59.37^a^	45.82^b^
Black plastic	68.71^a^	42.96^bc^
LSD(0.05)	12.15	
CV (%)	15.52	

Means followed by the same letters in a column and rows are not significantly different from each other according to the LSD test at 5 % level of significance.

**Table 9:** Means of total fruit yield of watermelon as affected by the interaction effect of mulches and varieties.

Treatments	Total fruit yield (t ha^−1^)	
Mulches	Varieties	
	Crimson Sweet	Sugar Baby
Control	35.84^d^	39.67^cd^
Grass	49.26^c^	44.02^cd^
White plastic	62.61^ab^	50.42^bc^
Black plastic	72.42^a^	47.22^cd^
LSD(0.05)	13.32	
CV (%)	15.15	

Means followed by the same letters in a column and rows are not significantly different from each other according to the LSD test at 5 % level of significance.

**Table 10:** Correlation between and among different crop growth and fruit yield parameters.

	MVL	NLB	NLMV	NNMV	NMFPP	NTFPP	AVFW	MFY	TFY	FL	FDI
**MVL**	1	0.92***	0.93***	0.80***	0.62**	0.36	0.63***	0.73***	0.67***	0.79***	0.80***
**NLB**		1	0.95***	0.69***	0.62**	0.25	0.57**	0.70***	0.63***	0.74***	0.79***
**NLMV**			1	0.66***	0.64***	0.31	0.56**	0.68***	0.60**	0.71***	0.72***
**NNMV**				1	0.54**	0.33	0.63***	0.69***	0.65***	0.87***	0.81***
**NMFPP**					1	0.81***	0.60**	0.66***	0.64***	0.47*	0.43*
**NTFPP**						1	0.47*	0.45*	0.48*	0.23	0.21
**AVFW**							1	0.97***	0.98***	0.66***	0.69***
**MY**								1	0.98**	0.73***	0.74***
**TFY**									1	0.68***	0.70***
**FL**										1	0.90***

*, ** and *** correlation is significant at 5 %, 1 % and 0.1 % probability levels, respectively. The decimal numbers without any asterisk are non-significant (*P* > 0.05). MVL= Main vine length, NLB= Number of lateral branches per vine, NLMV=Number of leaves on main vine, NNMV=Number of nodes on main vine, NMFPP=Number of marketable fruit per plant, NTFPP=Number of total fruit per plant, AVFW= Average fruit weight, MFY=Marketable fruit yield, TFY=Total fruit yield, FL=Fruit length, FDI=Fruit diameter.

**Table 11:** Partial budget analysis of watermelon yield as affected by mulches and varieties for watermelon Production.

Mulches	Variety	MY(t ha^−1^)	AjMY (t ha^−1^)	GI (ETB ha^−1^)	TVC (ETB)	NB (ETB ha^−1^)	MRR (%)
Control	Crimson Sweet	28.777	25.8993	388,489.5	18,516.2	369,973.3	–
	Sugar Baby	30.956	27.8604	417,906	20,323.6	397,582.4	1527.5
Grass	Sugar Baby	37.712	33.9408	509,112	83,404.97	425,707.03	44.6
	Crimson Sweet	43.574	39.2166	588,249	86,422.17	501,826.83	2522.8
White plastic	Sugar Baby	45.818	41.2362	618,543	233,130.69	385,412.31	D
	Crimson Sweet	59.369	53.4321	801,481.5	240,761.29	560,720.21	2297.4
Black plastic	Sugar Baby	42.957	38.6613	579,919.5	241,414.09	338,505.41	D
	Crimson Sweet	68.706	61.8354	927,531	256,363.49	671,167.51	2225.2

*Note:* In the year 2021 price of watermelon was 15 Birr kg^−1^; price of Crimson Sweet and Sugar Baby seed was 1250 and 1750 birr ha^−1^, respectively; price of grass mulch was 125 birr per plot; price of black and white plastic mulches was 40 and 38 Birr for 2m^2^ area, respectively; labor cost to apply grass and plastic mulches was 6944.4 and 13,889.9 birr ha^−1^, respectively. **ETB**= Ethiopian birr; **MY**=Marketable yield; **AjMY**= Adjusted marketable yield; **GI**= Gross income; **TVC**= Total variable cost; **NB**= Net benefit; **MRR**= Marginal rate of return, *D*= Dominated.

## 4 Experimental Materials, Design and Methods

Two open pollinated watermelon varieties were used in the trial. The seed of these varieties were obtained from Bakker Brothers Seed Company and selected based on production status and average performance in yield and quality and agro-ecological adaptation. White plastic, black plastic and thatch grass were used as mulching materials. The thickness of all plastic mulch was 30 µm while grasses were applied at 5 inch thick.

The treatments were factorial combination of two varieties of watermelon (Crimson Sweet and Sugar Baby) and four type of mulches (black plastic, white plastic, grass mulch and no mulch as control). The experiment was laid out as a randomized complete block design (RCBD) in a factorial arrangement with three replications. The experimental field was plowed using oxen plow and harrowed to make the soil fine tilth. Then the plots were measured, demarked and laid out. The mulch materials were laid down on the beds and small holes were opened at a spacing of 1.5 m between rows and 1 m between plants and three seeds of each variety were sown in each hole during the first week of January. There were 0.5 m and 1 m spacing between adjacent plots and each replicate or block. The plot size was 5 m × 3 m (15 m^2^). There were two rows on each plot which contained ten plants. Five plants were randomly selected from each experimental plot for recording observations and measurements leaving one plant at both sides of each rows as border plants. Then, all other intercultural practices such as gap filling, thinning, fertilizer, weeding, hoeing and training as well as plant protection measures against insect pests and diseases were done uniformly when required during the growing period as per recommendation.

## 5 Data collected

### 5.1 Crop phenological parameters

Days to 50 % emergence were recorded from the time of sowing to the date when 50 % of the plants appeared above the ground in each plot. Days to 50 % flowering were recorded as number of days taken from the date of sowing to the date 50 % of plants in each plot initiated flowers. Days to 50 % maturity were recorded as number of days taken from the date of sowing to the date 50 % of the plant in each plot has their tendrils closest to the fruit turned brown and shriveled and the fruits given out dull or a dead sound when thumped [4].

### 5.2 Growth parameters

Main vine length of plants were measured in centimeters from the base of the plant to the tip of the main stem at 30, 45, and 60 days after sowing (DAS) and final harvesting of the crop from five randomly selected plants per plot and the mean was used for the analysis. Number of lateral branches per vine were counted from five randomly selected plants per plot and the mean was used for the analysis. Number of nodes on main vine were counted from five randomly selected plants per plot at the time of final harvesting and the average was taken for the analysis. Number of leaves on the main vine were counted from five randomly selected plants per plot at 30, 45 and 60 days after sowing (DAS) and the average was taken for analysis.

### 5.3 Yield and yield component parameters

Number of fruits per plant were counted at each harvest and the total was recorded from a sample of five plants per plot. Number of marketable fruit per plant were recorded from well developed, tender and edible fruits which are free from diseases, insect pest, physical defects and fruits that weighed 1.7 kg and above [5] were counted from a sample of five plants per plot. Number of unmarketable fruit per plant was counted as number of fruit per plant which were diseased, sun- scalded, rot, insect attacked and small sized (< 1.7 kg) were counted and recorded [5]. Fruit length was measured from five randomly selected fruits after harvest in centimeter from the base of the fruit to the tip of the fruit using Vernier caliper. Fruit diameter was measured from five randomly selected fruits after harvest at the middle of the fruit using Vernier caliper and the average was expressed in cm. Average fruit weight was recorded in kilogram from five randomly selected fruits after harvest were weighed and the sum of weight of the fruits were divided by total number of fruits. The weight of fruits were measured by digital balance. Marketable fruit yield was recorded from well developed, tender and edible fruits which are free from diseases, insect pest and other physical effects and fruits that weighed 1.7 kg and above [5] were harvested and recorded and then converted into ton per hectare basis. Unmarketable fruit yield was determined by weighing the counted number of unmarketable fruits which were diseased, sun- scalded, rot, insect attacked and small sized (< 1.7 kg) [5] and expressed in ton ha^−1^. Total fruit yield were recorded as the sum total of marketable and unmarketable fruit yield in ton ha^−1^.

### 5.4 Statistical data analysis

The collected data were subjected to analysis of variance (ANOVA) using SAS statistical software version 9.4 software computer program's General Linear Model (GLM) procedure [1]. As described in Montgomery [2], the residuals were examined to verify the normal distribution and homogeneous variance model assumptions on the error terms for each response variable. Least significant difference (LSD) test at 5 % probability levels of significance was employed to separate treatment means where significant treatment differences existed. Linear correlation analysis among the different growth and yield parameters were made using Pearson correlation coefficient.

### 5.5 Partial budget analysis

The economic analysis was calculated to determine the best combination of variety and mulch type. Data were collected on price of mulches used, labor cost for application of mulches, seed costs and the price of marketable fruit yield of watermelon after harvest were taken into account in order to finally come up with a recommendation which can result in highest benefit cost ratio. This will determine whether or not the benefits outweighed the costs and by how much.

Therefore, for economic evaluation of the cost and benefit in using varieties and mulches, the partial budget analysis which includes the Dominance Analysis (DA) and Marginal Rate of Return (MRR), was used following the CYMMYT procedure [6]. Marketable fruit yield and economic data were computed to compare the advantage of using varieties and application of mulches. As an output, total gross benefit was calculated from marketable fruit yield of watermelon. Local market price of watermelon fruit was assessed during the harvest time and was changed to hectare bases.

From the result of this study, the average yield of eight (8) treatment combinations was obtained. According to [6], the average yield was adjusted down wards by 10 %. This is for the reason that, researchers have assumed that using the same treatments the yields from the experimental plots and farmers’ fields are different, thus average yields should be adjusted downward. Based on this, the recommended level of 10 % was adjusted from all treatments to get the net yield. In addition to this, to obtain the gross field benefits, it was essential to know the field price value of one kg of watermelon fruit during harvesting time. Then, the gross returns were computed by multiplying average market rate with the yield of respective treatments during the crop harvesting period. Deducting the total cost of production from this value gives the net income and the net income is divided by the total cost (cost of mulches, seed costs and labor cost to apply mulches) to obtain the benefit cost ratio and the ratio of changes in net income and total cost to obtain MRR. Finally, the treatment with the respective mulching types that gave the maximum benefit cost ratio and net return were selected as presented in Table 11.

## Limitations

None of problems faced during data collection, curation or size of dataset.

## Ethical Statement

The dataset collected in this study did not involve animals and humans.

## CRediT authorship contribution statement

**Getahun Yismaw:** Conceptualization, Methodology, Software, Writing – original draft, Investigation, Formal analysis. **Solomon Fantaw:** Supervision, Writing – review & editing. **Asrat Ayalew:** Supervision, Writing – review & editing.

## Data Availability

Data on Effect of Mulches on Growth and Fruit Yield of Watermelon (Citrullus lanatus Thunb.) Varieties in West Dembia District, Central Gondar Zone, Ethiopia (Original data) (Mendeley Data)
